# The Role of Daylight for Humans: Gaps in Current Knowledge

**DOI:** 10.3390/clockssleep2010008

**Published:** 2020-02-28

**Authors:** Mirjam Münch, Anna Wirz-Justice, Steven A. Brown, Thomas Kantermann, Klaus Martiny, Oliver Stefani, Céline Vetter, Kenneth P. Wright, Katharina Wulff, Debra J. Skene

**Affiliations:** 1 Sleep/Wake Research Centre, Massey University Wellington, Wellington 6021, New Zealand; 2 Centre for Chronobiology, Psychiatric Hospital of the University of Basel, 4002 Basel, Switzerland; anna.wirz-justice@unibas.ch (A.W.-J.); Oliver.Stefani@upk.ch (O.S.); 3 Transfaculty Research Platform Molecular and Cognitive Neurosciences (MCN), University of Basel, 4002 Basel, Switzerland; 4 Chronobiology and Sleep Research Group, Institute of Pharmacology and Toxicology, University of Zürich, 8057 Zürich, Switzerland; steven.brown@pharma.uzh.ch; 5 Faculty for Health and Social Affairs, University of Applied Sciences for Economics and Management (FOM), 45141 Essen, Germany; thomas.kantermann@fom.de; 6 SynOpus, 44789 Bochum, Germany; 7 Psychiatric Center Copenhagen, University of Copenhagen, Rigshospitalet, 2100 Copenhagen, Denmark; Klaus.Martiny@regionh.dk; 8 Department of Integrative Physiology, University of Colorado Boulder, Boulder, CO 80309, USA; Celine.Vetter@Colorado.EDU (C.V.); Kenneth.Wright@colorado.edu (K.P.W.J.); 9 Division of Endocrinology, Metabolism and Diabetes, University of Colorado, Aurora, CO 80045, USA; 10 Departments of Radiation Sciences and Molecular Biology, Umeå University, 901 87 Umeå, Sweden; katharina.wulff@umu.se; 11 Wallenberg Centre for Molecular Medicine (WCMM), Umeå University, 901 87 Umeå, Sweden; 12 Chronobiology, Faculty of Health and Medical Sciences, University of Surrey, Guildford GU2 7XH, UK; d.skene@surrey.ac.uk

**Keywords:** natural light, spectrum, twilight, circadian rhythms, melatonin, entrainment, health, sleep, alertness, mood

## Abstract

Daylight stems solely from direct, scattered and reflected sunlight, and undergoes dynamic changes in irradiance and spectral power composition due to latitude, time of day, time of year and the nature of the physical environment (reflections, buildings and vegetation). Humans and their ancestors evolved under these natural day/night cycles over millions of years. Electric light, a relatively recent invention, interacts and competes with the natural light–dark cycle to impact human biology. What are the consequences of living in industrialised urban areas with much less daylight and more use of electric light, throughout the day (and at night), on general health and quality of life? In this workshop report, we have classified key gaps of knowledge in daylight research into three main groups: (I) uncertainty as to daylight quantity and quality needed for “optimal” physiological and psychological functioning, (II) lack of consensus on practical measurement and assessment methods and tools for monitoring real (day) light exposure across multiple time scales, and (III) insufficient integration and exchange of daylight knowledge bases from different disciplines. Crucial short and long-term objectives to fill these gaps are proposed.

## 1. Introduction: The Need to Identify Current Gaps of Knowledge in Daylight Research

Our rotating planet, with its alternating 24-h light–dark cycles, changing day lengths with seasons, and weather conditions, has major effects on the physiology and behaviour of organisms from prokaryotes to mammals. One adaptive response to this dynamically changing geophysical environment in most organisms has been the evolution of biological time-keeping systems, i.e., biological “clocks” capable of synchronisation (entrainment) to environmental time cues, providing the enormous advantage of anticipation to environmental changes, allowing corresponding adaptations in behaviour (e.g., shelter, reproduction) and physiology (e.g., metabolism, quiescence). Since the early 1980s, it is known that many aspects of biological clock function are highly conserved across species, for example, the intracellular structure of clocks themselves, a feedback loop encoded by dedicated “clock genes” first described in fruit flies [1,2] and later in mammals [3]. Another highly conserved evolutionary mechanism is (day-) light perception via specialised light-sensitive molecules in specific cells on the outer surface of multicellular organisms. Within the animal kingdom, opsins are the common photopigment molecules of all visual systems [4,5]. A unique ancestral photopigment melanopsin—highly conserved and considered the evolutionary linkage between invertebrate and vertebrate photopigments—plays a special role in so-called nonvisual light perception, including circadian photoentrainment [6,7,8]. The intrinsically photosensitive properties of melanopsin were first described in frog skin [9], and later in mammalian eyes [10,11,12], including humans [13]. These examples emphasise the fact that important time-keeping processes supporting adaptation to the natural day–night environment evolved over a large time scale (millions of years).

Only very recently on this time scale, i.e., less than 200 years ago, technical progress—the discovery of electric lighting (and electric power)—enabled, for the first time, relative independence from (natural) daylight. Even though this invention has been of great benefit for humankind, it has also thoroughly and irreversibly changed our home, social and work environments. There are two main reasons for these changes: Firstly, relative independence from the natural light–dark cycle fostered 24/7 work and living conditions throughout the developed world. Secondly, the use of electric lighting has not only been restricted to night time but has also made it possible to live and work independently of daylight during the day. There is now growing evidence that these changes have had broad consequences for health^1^ issues worldwide (see, e.g., [14]). 

One aspect of these health consequences relates to inadequate “entrainment” of biological clocks to electric light. Entrainment is an active process by which environmental time cues, zeitgebers (German for “time cue”), such as the light–dark cycle, produce stable and appropriate timing of internal biological clocks [15,16,17]. Within the biological rhythm research community, there are concerns about the weakness of zeitgeber strength for circadian entrainment in electrically lit environments (with no or low natural daylight exposure). Circadian disruption, as a consequence of inadequate entrainment, could impact on robustness, regularity, and amplitude of biological rhythms in individuals and within different populations [18]. Adverse health effects, in which the lack of sufficient daylight has been directly (daily light and dark exposure) or indirectly (via circadian misalignment) involved, range from sleep problems and impaired daytime functioning to increased prevalence of chronic diseases such as depression, obesity, diabetes, cardiovascular diseases and cancer [19,20,21,22,23,24,25,26]. 

The contribution of circadian rhythm disruption to disease pathogenesis is likely to be multifactorial. In other words, when work, social and environmental pressures disrupt the entrainment of circadian rhythms by light and non-photic zeitgebers (food, physical activity), the out-of-sync circadian physiology may predispose to altered stress levels and lifestyle changes (fatty diet, alcohol consumption, smoking, self-medication, lack of exercise, short sleep). Together, this will alter multiple brain and body circuits that underpin physical and mental health, representing a multifactorial basis for any form of pathophysiology [27,28].

Scheduled daylight and mixed daylight/electric light exposure conditions may also optimise the effectiveness of certain medications/drugs and reduce side effects directly or indirectly via inducing robust circadian rhythmicity [29]. The latter would enable timed coordination between the kinetics of a drug’s actions and the host’s responsiveness. However, we are just at the beginning of understanding these points, and replication and further larger-scale studies are warranted. 

To some individuals, the idea that the light environment has the potential to significantly influence human health outcomes may seem exaggerated. However, this is long established in the commercial application of light (photoperiod, intensity, and spectral balance of wavelengths) in growing plants, flowers, fruits, poultry and livestock [30], though with emerging negative consequences from climate change [31]. Humans may similarly require exposure to specific light patterns for optimal physiological and psychological well-being. 

Given the primordial role of light for the circadian system and other non-visual functions and the regulatory role of the circadian system on a multitude of physiological processes, it is crucial to identify (and address) current gaps in daylight research. In this report, we use the term “daylight” to comprise mixed daylight/electric light conditions, which is thus implicitly included throughout the manuscript if not stated otherwise. With respect to health, daylight research should also include the development of coping strategies against chronically altered (day-) light conditions, such as experienced in atypical work schedules, including shift work. Daylight research may also elucidate how adequate daylight exposure could maintain health, prevent disease, and reduce circadian disruption. The main goals of daylight research in a human context are thus: (1) To elucidate the effectiveness of daylight to promote general health, quality of life and foster coping strategies. (2) To develop standardised tools to accurately and continuously measure daylight (and electric light) as well as methodologies to determine its effects on visual, psychological, and somatic functions. (3) To translate existing knowledge about human responses to daylight into appropriate designs for daylight-enhanced buildings and urban settings at the planning stage. This paper addresses these three goals by identifying main knowledge gaps, defining related, unanswered questions, and suggesting possible ways to fill these gaps and develop potential coping strategies. 

To identify gaps of knowledge in daylight and to address them is complex because the implications of daylight go far beyond isolated and specialised research areas; they impact broadly on many disciplines such as science of the built environment (physics, engineering, architecture), environmental sciences, medicine, psychology, economics, occupational and social sciences. The unanswered questions related to daylight research are those which have been explored under electric light conditions (mostly in the laboratory) but have not yet been answered for daylight research (which is mainly related to daylight inside buildings). There are questions that are specific to daylight and cannot be (easily) mimicked by electric light (e.g., seasonal changes in day length or twilight conditions). Also, some questions and problems have to date not been addressed either with daylight or electric light. Our report is designed to highlight some of these questions and put daylight into the focus of research. Obviously, quantities and qualities can vary considerably between daylight and electric light, especially inside buildings, and to date, it is unknown whether daylight is superior to electric lighting with respect to the impact on humans (and if so why). 

This paper was developed from an interdisciplinary workshop involving members of the Daylight Academy (DLA; www.daylight.academy) in November 2017 in Lausanne (Switzerland). The “missing links” in defining the biological value of daylight (and darkness at night) for human beings were discussed with respect to vast regional differences in daylight availability across the year from the equator to high latitudes. A second meeting took place in June 2018 in Berlin (Germany) to discuss the role of daylight and specify gaps and barriers to the advancement in knowledge. Therefore, the workshop outcome is obviously primarily focused on daylight. In addition, specific action points were named to achieve the goals. These goals include evidence-based knowledge-transfer for enabling practitioners to design living and working spaces and conditions in a regional-adapted manner for the benefit of the inhabitants. 

We do not systematically review the literature on the current state of daylight research or carry out a meta-analysis. Since this paper is an enlarged output of two workshops on daylight and its impact on humans, it differs in its formal structure from an experimental study. 

**Figure 1:**
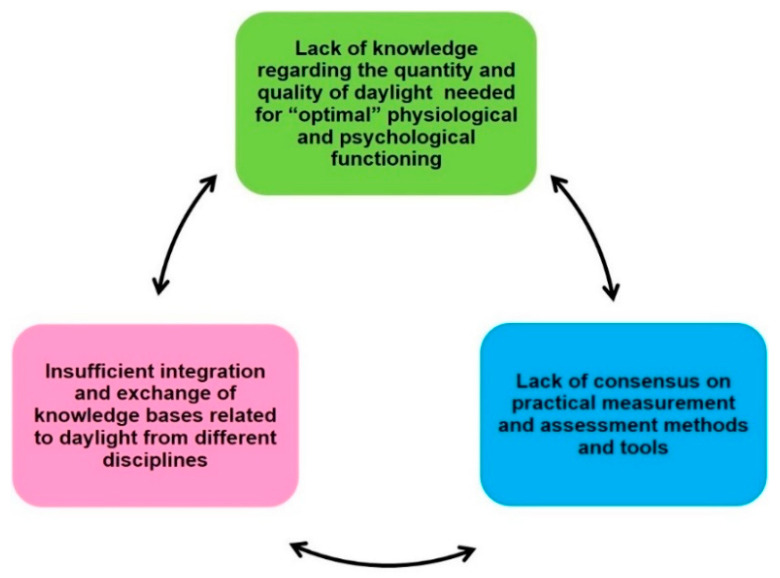
The identified three main groups of gaps of knowledge in daylight research.

This report is envisaged as a framework for stimulating interdisciplinary research and collaborative approaches to the neglected area of daylight research in humans. During the DLA workshops, we came up with a list of gaps, which were retrospectively assigned to three main groups (Figure 1): (I) Lack of knowledge regarding the quantity and quality of daylight needed for “optimal” physiological and psychological functioning (optimal = relating to best possible quality of life for an individual); (II) Lack of consensus on practical measurement and assessment methods and tools for real (day-) light exposure across multiple time scales, especially under long time recording periods (e.g., circadian, seasonal contexts); and (III) insufficient integration and exchange of knowledge bases related to daylight from different disciplines. This grouping is not based on any *a priori* concept, but rather grew out of the two workshops and meetings within the DLA. A summary of the many *physical* differences between the properties of daylight and electric light have been recently published by members of the DLA [30,32].

## 2. Gaps Group I: Lack of Knowledge Regarding Quantity and Quality of Daylight Needed for “Optimal” Physiological and Psychological Functioning

We live in a global and increasingly digitised 24/7 society. Among the key gaps in daylight research is the lack of knowledge about timed combinations of quantity and quality of daylight (including mixed day/electric light conditions) required by a given individual each day. This information is necessary to cope with and mitigate challenges arising from electrically lit life and work conditions. The term “optimal” hereby refers not to an absolute value, but rather to the best possible quality of life for an individual under his or her environmental conditions. Lighting standards for the visual system have been developed; here, we focus primarily on the non-visual physiological and psychological functions. This first group of gaps in daylight research contains a variety of different subgroups: (Section 2.1) vision and visual comfort; (Section 2.2) physiology and behaviour; (Section 2.3) circadian entrainment; (Section 2.4) “optimal” (day-) light dose; (Section 2.5) light sensitivity; (Section 2.6) therapeutic aspects; (Section 2.7) risks; (Section 2.8) inter-individual differences; (Section 2.9) work conditions; and (Section 2.10) environmental factors.

### 2.1. Open Questions for the Effects of Daylight on Vision and Visual Comfort

The effects of electric light on vision (e.g., [33,34]), or light conditions for vision at workplaces (e.g., [35]) were extensively explored many decades ago, but it is still not clear whether there are daylight-specific influences on vision, visual capabilities, visual processing and comfort, which may arise from the physical differences between daylight and electric light [32]. More specifically:

- Daylight impacts on vision and visual comfort with respect to colour perception [36], colour preferences (e.g., [37]), as well as homogeneity, dynamics, glare and flicker from mixed daylight/electric lighting conditions (e.g., [38]).
- There is considerable literature on visual comfort and glare evaluations with daylight inside buildings, e.g., [39], but very little is known about mixed/synergistic effects of high visual comfort together with non-visual functions such as alertness, mood [40,41,42] and indoor temperature perception [43,44]. These mixed/synergistic effects are missing not only for daylight but also for mixed electric/daylight conditions.

### 2.2. How Does Daylight Impact on Physiology and Behaviour Beyond Vision?

There is now a large body of evidence from human studies on the non-visual physiological and psychological effects of electric light under controlled laboratory conditions (e.g., [45,46,47,48,49,50,51,52,53,54,55,56,57,58,59,60,61,62]). An increasing number of studies have also investigated the non-visual effects of daylight alone or with mixed daylight/electric light (e.g., [43,63,64,65,66,67,68]), which better reflects the situation at most work and residential places. A big difference with daylight is, of course, that there is also usually a view, i.e., being outdoors or looking out of the window into green (or built) spaces, to the sky, which per se is positively rated [69,70,71]. Some examples of knowledge gaps in daylight research for five non-visual functions are shown in Table 1.

### 2.3. Gaps of Knowledge on Circadian Entrainment

Most chronobiological research in humans over the past 50 years has mainly dealt with (controlled) electric light in the laboratory, “bunkers” or caves [90,91,92,93,94,95,96,97]. The biggest issue here is certainly removal from any natural photic context and reduced external stimuli. These studies were necessary to understand what we currently know, but now it is time to move to more naturalistic approaches, including studying daylight, and/or mixed electric light/daylight during the daytime [98]. Surprisingly, there are only a few studies looking at circadian entrainment in humans under natural conditions [78,79], and most of them have been short-term studies. There is evidence from a few isolated populations, still living without electric lighting, that the circadian phase of entrainment relative to the external light–dark cycle is indeed different from that in regions supplied with electric light sources [99]. There is a considerable gap of knowledge about the impact of natural light under real-life conditions, as summarised in Table 2.

### 2.4. What Is the “Optimal” Dose of Daylight with Respect to Intensity, Spectrum and Timing?

From laboratory studies with electric lighting interventions during the daytime, mixed dose-response effects have been reported for intensity, duration and spectral composition: Some studies found effects of daytime/evening electric light exposure on subjective and/or objective variables [52,86,89,115], while others did not [82,116]. Few studies have been conducted under daylight conditions only or mixed daylight/electric light conditions, and most of these are applied or semi-natural studies [40,65,78]. The general unanswered questions are related to daylight thresholds, duration/intensity and spectral responses, as well as the role of the dynamics of daylight. To date, these complex daylight properties have not been evaluated in a single model. There is evidence of a complex interaction between different subtypes of ipRGC (modulated by rod and cone input), which depend on the daylight spectrum and irradiance [117]. Finally, the main question also remains—why should daylight be different from findings with electric light? The outstanding questions are summarised in Table 3.

### 2.5. How Does Daylight Influence Light Sensitivity Functions? 

There are a few field and laboratory studies showing indirectly that sensitivity to light increases when there is a lack of daylight or predominantly low indoor light exposure [126,127,128,129,130], but there is no consensus how to measure light sensitivity nor about the length of daylight nor the mixed electric light/daylight exposure necessary to counteract such increased sensitivity. This is of great importance since chronic exposure to low daylight levels during daytime may increase vulnerability to light in the evening/night due to increased sensitivity, as shown under electric light [80,131,132].

Another aspect of daylight and retinal functions is the question of underlying mechanisms and counterstrategies for the recent high increase of myopia cases in children (e.g., South East Asia). Growing evidence suggests that chronic low daylight exposure is one of the possible triggering factors; for a recent review, see [133].

### 2.6. Is Daylight Exposure Effective as a ‘Treatment’?

There is clear evidence for the therapeutic use of controlled bright and/or dynamic light exposure (with or without daylight) in hospitals and nursing homes [134,135,136,137,138], as well as light therapy lamps for a variety of psychiatric (e.g., seasonal and non-seasonal affective disorder, bipolar depression [123,124,125], ADHD [139], borderline personality disorder [140]), neurologic (Parkinson’s disease [141,142]), and medical disorders to improve sleep (e.g., [143]) or reduce fatigue [144]. Only a few studies have explored scheduled daylight exposure for therapeutic use to obtain information about duration and timing as related to improvement [145,146,147,148].

The question is why not use daylight instead of light therapy lamps since daylight is freely available and without energy costs, and there are other beneficial effects with daylight exposure such as a view, contact with nature, other people? There is, at present, insufficient evidence to replace light therapy lamps with timed daylight as treatment for the above indications. It would be important—and practical—to know if daylight is equally effective or even superior to electric light as therapy. One study in seasonal affective disorder suggested outdoor light is equivalent in response [145]. We need to know if regular daylight exposure or mixed electric light/daylight (and darkness at night) enhances recovery/promotes health in hospitalised patients more efficiently (e.g., post-surgery, intensive care [149,150,151]), and in healthcare institutions (e.g., psychiatric wards, nursing homes [152,153]) than with electric light alone. The question arises as to which daylight interventions are needed for which groups (e.g., patients with impaired vision/visual blindness, circadian rhythm sleep–wake disorders, depression and other psychiatric/neurologic diagnoses), adolescents or older people, shift workers, men and women? 

### 2.7. Are There Risks of Daylight Exposures (e.g., Systemic Diseases, Dermatology, Ophthalmology)?

Much is known about the direct UV risk from sunlight for skin and eyes in humans, and current regulations are regularly evaluated [154]. National programs to reduce skin cancer have been launched worldwide. There is, however, only very little evidence as to whether having full sun protection all the time is beneficial for other physiological functions. A recent study (performed in South Asia) showed that wearing a sun hat and sunglasses while being outside still allows a good portion of daylight to be transmitted via the eyes [155], but this finding also needs to be shown for places further from the equator.

In addition: How can light-sensitive populations be protected from too much (day-) light (e.g., porphyria patients, patients taking light-sensitising medications, photophobic patients, patients with migraine, certain patients with retinal damage, patients with skin diseases), whilst still retaining enough exposure to ensure proper/adequate functional outcomes (e.g., vitamin D synthesis, sleep quality, waking functions, circadian entrainment and general health)?

### 2.8. Gaps of Knowledge Related to Individual Differences

We know of some factors that can explain inter-individual differences in response to light exposure, including age, chronotype, gender, genotype and many more. However, there is much more unknown, and it is not clear whether these inter-individual differences are manifested under daylight conditions. Here (Table 4), we list some of the known inter-individual “trait” differences mainly in response to electric light, which also need to be considered for daylight research (and where the mechanisms remain to be elucidated). Some of these inter-individual differences are only known for non-human species and have not yet been explored in humans. Beyond these inter-individual traits, it might also be important to know what the consequences of such differences are—for example, sex differences in the perception of light of a special quality might lead to implications on the societal aspect of gender for light applications.

### 2.9. Gaps of Knowledge in Daylight Research for Work Conditions

Many field studies have tested different electric light scenarios at work, but from most of the studies, it is not clear if and how daylight exposure in addition to electric light was considered for the analysis; therefore, many questions remain. In addition, many aspects related to work conditions overlap with general aspects, as mentioned in Section 2.1, Section 2.2, Section 2.3, Section 2.4, Section 2.5, Section 2.7 and Section 2.8. Table 5 summarises some specific work and daylight associated questions:

### 2.10. Gaps in Knowledge of Daylight Research Related to Environmental Factors

In our built environment, many factors influence the quantity and quality of daylight exposure. These mostly physical factors are per se well known, but the interactions with and consequences on humans of latitude, time zones, micro- and macroclimate, architecture and urban settings are only poorly understood (Table 6). Therefore both the spectrum and intensity of the daylight influx which reaches indoor rooms matters, and is, of course, dependent on architectural properties, building orientation, windows size, glazing, and geographical latitude. And lastly, how relevant is the concept of the “biophilia” hypothesis, which describes that humans tend to find connections with nature and living organisms, in this context (e.g., [206])?

## 3. Gaps Group II: Lack of Consensus on Practical Measurement and Assessment Methods and Tools for Monitoring Real (Day-) Light Exposure Across Multiple Time Scales

This crucial group of gaps describes the lack of appropriate methods in basic and applied (day)-light research. Many of these gaps apply generally to lighting research. The most important of these gaps are summarised in Table 7.

## 4. Gaps Group III: Insufficient Integration and Exchange of Daylight Knowledge Bases from Different Disciplines

**Figure 2:**
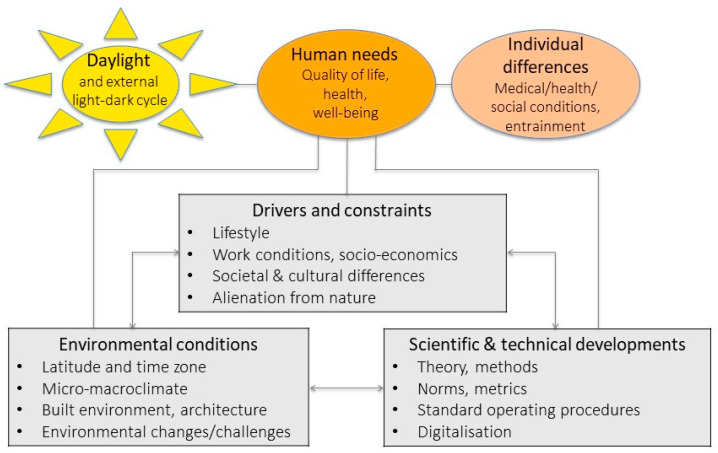
Illustration of daylight and other factors as determinants of human health. There are two main dimensions at the individual level, which are impacted by daylight: human needs and individual differences. There are three dimensions of reciprocal actions that modify the impact of daylight on humans: (1) drivers and constraints, (2) environmental conditions and (3) scientific and technical developments.

The impact of daylight depends on many “human” factors but is also determined by environmental and technical aspects and interacts with a variety of drivers and constraints at different levels and dimensions (see Figure 2). As such, daylight research cannot be comprehensively done exclusively within a single research community; it must involve many disciplines. A fundamental key gap is related to insufficient transdisciplinary approaches, with greater participation of a broader range of experts from different disciplines such as physicists (simulation modelling), ecologists, landscape architects (ecological laws), engineers (glazing, building principles), biomedical disciplines (health), biologists and psychologists (physiology and behaviour), artists, media/journalists, social scientists (social-ecology connectedness), architects and economists, to be able to find solutions for the connectedness between daylight/nature/living space/humans.

Access to natural daylight is nowadays unfortunately not considered as an explicit aim by leading institutions worldwide, such as, for example, the WHO, where the “Healthy Cities” concept, although it mentions access to green spaces, does not explicitly require access to daylight [226]. Accessibility to daylight should be part of global discussions about sustainable living, health and well-being, and should be included in the UN 2030 Agenda for Sustainable Development and the WHO’s “health for all” policy [227].

## 5. What Is Needed to Fill These Gaps and Achieve the Goals in Daylight Research? 

The need to develop new tools, methods and approaches seems crucial, and here we highlight some possibilities. Table 8 and Table 9 summarise the short- and long-term objectives in daylight research, respectively.

## 6. Summary

Daylight research is much more complex and less controllable in the field than pure electric light research in the laboratory, and thus scientific methods and tools need to be developed to make daylight research accessible and studies comparable. Even more important is agreeing on methodology (SOPs) and running collaborative projects to provide large data sets. 

## 7. Highlights

- Recognising the importance of daylight for human health and well-being.
- Daylight research needs to define requirements for optimal physiological and psychological functioning.
- New techniques are required to monitor and assess (day-) light exposure in the field.
- Interdisciplinary exchange of daylight knowledge is the key to integrating findings into practice, whether architectural or medical.

## Figures and Tables

**Table 1:** Some open questions in mixed electric light/daylight research related to metabolic functions, sleep, alertness and cognition as well as physical activity.

**(i) Metabolic functions**	
How does electric light and/or daylight affect metabolic functions?	This question is closely related to meal timing, caloric intake and meal composition, or weight loss. A few studies have investigated these aspects under laboratory conditions [72,73,74], but not yet under daylight conditions. The question is whether daylight exposure specifically affects the timing of meals, post-prandial responses, temperature regulation, metabolism, body composition and the gut microbiome?
**(ii)** **Sleep**	
Does daylight and/or mixed daylight/electric light conditions during the day mediate better sleep quality at night, and if yes, how?	There is some evidence that indoor and/or outdoor bright light exposure during the day leads to longer sleep duration and increases sleep quality as was shown in laboratory and field studies [63,64,75]. Electric light exposure (with a spectral power distribution closer to daylight than standard LED-light) during daytime enhances slow wave sleep (=‘deep sleep’) [76] and brighter ambient light (compared to dim light) increased homeostatic sleep pressure during wakefulness [77]. The following questions relate to daylight and sleep: Does insufficient sleep (partial and/or chronic sleep deficit) counteract beneficial daylight exposure effects? Can sufficient daytime light exposure offset negative consequences of electric light exposure at night—with respect to entrainment, sleep, performance and health outcomes [78,79,80]?
**(iii) Alertness and** **cognition**	
How do daylight-specific properties affect alertness and cognitive functions?	Many laboratory studies with steady state electric lighting showed light-dependent alertness [45,46,49,81,82,83] and cognitive repercussions [46,53,55]. A few studies have looked at spectrally tuned electrical light conditions and alertness and cognition [76,84,85,86].
**(iv)** **Physical** **activity**	
Does physical activity and daylight interact to induce larger phase shifts of the circadian clock?	From studies in the laboratory there is a phase-response curve of physical activity with both phase delays and advances at specific times of day [87]. Activity facilitates phase delays in very dim light [88]. The question relates to the interaction of exercise with (day-) light exposure as shown with cycling performance [89].

**Table 2:** Gaps in knowledge about the impact of natural light under real-life conditions.

**Daylight and Circadian Entrainment**	
**(i) Daylight properties:** Quality/quantity	Which combinations of daylight qualities and quantities are relevant for circadian entrainment (alone and in combination with electric light sources) and dependent on time of day?
Spectrum/colour	Which frequency bands of the electromagnetic spectrum are relevant when and how important is the integration of colour [100]?
Dynamics	Do ultradian variations/dynamics of daylight play a role in circadian entrainment?
Twilight	What is the role of twilight (dawn and dusk) for circadian entrainment in humans [101,102]? For example, is the colour signal of the “blue hour” as important for humans as it is for mice [103,104]?
Polarisation of daylight	What is the role of polarised daylight? Direct sunlight is not polarised but daylight from a particular region of the sky is partially polarised. In contrast to bees, humans can hardly perceive polarised light, however a physiological influence of polarised light on humans cannot yet be excluded. A study by Brainard et al. showed no difference between unpolarised and polarised light on melatonin suppression [105].
Role of light distribution in the visual field, direction of light	A further characteristic of daylight is its large-area expansion with an unobstructed view of the sky. Non-human primates have ipRGcs distributed over the entire retina with a density of 3-5 cells/mm², and a maximum concentration of 20-25 cells/mm² around the fovea [106]. Since rods, cones and the ipRGCs in the eye are distributed over large areas of the retina, it is assumed that the non-visual effect of light is greatest when the light comes from a large source, such as an indirect illumination of a large bright area. In nature, this light comes from the sky. If only a small area of the retina is illuminated, as is the case with directed light from a spot, a weaker non-visual effect is assumed. The direction of light seems to be crucial at least for electric light that was shown to affect melatonin suppression [107,108,109] and sleep [110].
Season, latitude and day length	Does photoperiod length impact on circadian entrainment? From the literature it is known that there are differences in humans under purely natural seasonal lighting conditions when compared to mixed natural/electric light conditions [78,79,111].
**(ii) Effect on peripheral tissues**	How does electric light and/or daylight (indirectly) influence peripheral clock tissue function (e.g., metabolism, immune function, cardiovascular function, cell repair, detoxification, mitochondrial turnover)?
**(iii) Circadian misalignment/disruption**	How can electric and/or daylight help to mitigate circadian misalignment/circadian disruption (e.g., in shift workers [112,113,114], adolescents)?

**Table 3:** Unanswered questions related to properties of daylight and or electric light indoor lighting conditions.

Spectral range	Does the fact that the spectral power distribution of daylight goes beyond the visible range make a difference for non-visual functions, when compared to electric light? For example, the infrared (IR) portion that is always present in daylight is non-existent in electric light (except for banned incandescent light sources). It might have an important role for the retina, since a large body of literature suggests connections between long-wavelength radiation and (beneficial) physiological functions in the retina (see e.g., [118,119]). In addition, there is probably a specific role of the spectral power distribution and colour of twilight, as shown in rodents [103,104].
Duration and thresholds	- What are daylight or mixed electric/daylight exposure durations [52,120] and thresholds for non-visual functions, under real life circumstances? - What is the threshold of daylight/electric light conditions in the evening/night that does not interfere with sleep onset/propensity?
Dose-response relationships	What are the dose-response relationships for daylight and mixed daylight/electric light exposures for non-visual functions? In particular, with regard to the interaction of circadian and homeostatic processes with environmental conditions – that include electric light.
Irradiance and spectral composition	Daylight is spatially and temporally variable. These changes take place over very wide frequency bands: - What are these frequency bands, irradiances and are these variations required for the circadian system and other non-visual functions? - What is the influence of different contrasts of daylight or mixed electric light/daylight for photoreceptors (i.e., high vs. low melanopic irradiance) as was shown for electric light on visual perception [121], pupil light reflex and alertness [84,86]?
24 h-Dynamics	How do the 24-h dynamics of daylight impact on non-visual functions?
Light history	How can prior light history (see Table 4) of any light exposure be incorporated into the above-mentioned outcomes?
Therapeutic use of light	Light treatment has been established for winter and other depressive disorders and circadian sleep disturbances [122,123,124,125]. What should the daylight exposure recommendations be for these different therapeutic interventions (regarding intensity, spectrum, timing and prior light exposure)?

**Table 4:** Inter-individual trait and state differences in response to electric light or mixed electric light/daylight.

**(i) Physiological differences:** - Light sensitivity in adults [156,157,158] and children/adolescents [159,160,161], and elderly (less light transmission with age due to e.g., yellowing of the lens, cataracts [162,163,164,165], reduced pupil size [163,166]), Pupil responses to light in healthy persons [167,168,169,170] and patients with eye disease [171,172,173,174,175] Light responses depending on generalised medical and psychiatric status [176,177,178,179] and medication [180]
**(ii) Genetic differences:** - Known for the clock gene PER3 [181] and epigenetic modulators [182] - Genetic missense variant for melanopsin gene in seasonal affective disorder patients [183] - Melanopsin gene polymorphism [184] revealed changes in light sensitivity- Is there genetic adaptation of light sensitivity to geographical latitude, as was suggested for a polymorphism in PER3 gene length depending on latitude [185]?
**(iii) Cultural, behavioural differences:** - Cultural and social differences (e.g., clothing) [186,187] - What are the societal and ethnic/cultural differences in outdoor-related behaviour [188]? - How do various climate constraints limit going outdoors [186]?
**(iv) Mixed physiological/behavioural effects:** - Different photic histories, due to inter-individual differences in sleep/wake patterns [158,189] and/or - work/social schedules [126,128,190,191,192] (see also below – morning/evening types), - Sex/gender [193,194] - Does (day-) light exposure during pregnancy and early postnatal periods play a role in individual eye development - and circadian behaviour as shown in mice and rats [195,196]? - Morning/evening types [197,198,199] (i.e., chronotypes [200,201]—which may be considered a behavioural trait or as - proxy for phase of entrainment) and their preferences/choices for light [67,202,203]

**Table 5:** Identified workplace daylight as well as mixed electric light/daylight associated questions.

Daylight conditions for individuals at workplaces	How much and which qualities of daylight do different individuals/groups receive at their work- and living places (24/7), and how does this relate to their health status?
Daylight exposure as a countermeasure (for shift workers)	Can daylight be a means to counteract the detrimental effects of “light at the wrong time of day”, such as occurs with light in the evening or with (night) shift work? The reason might be that light during the day has a desensitisation effect for light exposure in the evening (see Table 3 and Table 4: light history), and in night shift workers bright light exposure after daytime sleep could help readjustment to the daytime work hours [204], see also recent recommendations of the ‘Working Time Society’ (WTS/ICOH; [113]).
Daylight and visual comfort and non-visual functions at workplaces	Are existing workspace regulations on glare and visual comfort at workplaces sufficient to concomitantly provide good and ‘biologically relevant’ daylight conditions [192]?
View/window	How important is the view out of the window and the environment outside the window [205] for workplace-related variables such as performance and alertness?

**Table 6:** Gaps in knowledge of daylight research related to environmental factors.

**Impact** **of latitude, time zones, climate**	
Seasonal changes	Does exposure to seasonal changes in day length have consequences for human physiology and health, as some of the existing literature suggests [79,111,207,208]?
Latitude	In those most vulnerable e.g., living at high latitudes, light therapy and improved home/work lighting has been shown to be useful for winter depression and sleep disorders, given that there is insufficient daylight for some months of the year [209,210].
Location within time zone	Does the location within a time zone modify daylight’s effect on an individual’s circadian phase [208,211]?
Daylight saving time (DST)	Is there a long-term effect of daylight saving time [212,213,214,215] on wake and sleep physiology?
Micro/macroclimate	How does ambient temperature, humidity, and air pollution modulate daylight’s effects on physiology?
Rural vs. urban environments	Is there a difference in daylight exposure dose between those living in rural vs. urban environments?
**Architecture, urban design**	
Daylight conditions in buildings	What role do the complex daylight conditions in buildings, such as building orientation, window positioning, glazing, play on visual comfort/adversity (glare), productivity and performance, circadian entrainment and health in general [32,216,217]? Could architecture be deployed to accentuate spatial-temporal modulation of daylight to stimulate retinal photoreception?
Glazing	How do single vs. double vs. triple glazing systems, electrochromic windows, and new technologies such as smart windows with integrated micro-daylighting systems influence health [218]? How effective is dynamic glazing in order to address heat emission and light?
Floor orientation, spatial distribution of daylight	How does the location of apartments (floor and geographical orientation) within a given building, and the indoor spatial distribution of daylight, affect human health?
Daylight enhanced qualities	What design can provide daylight-enhanced qualities in single buildings and urban settings?
Complementation with electrical light/daylight systems	How can the geographical orientation of a building, which determines its indoor light intensity, be compensated/complemented with daylight systems/electric light?
(Day-) light control systems	How should modern (light) sensor and controller technology be used to support health, performance, and well-being of its inhabitants?
Self-control of (day-) light	How much self-control over sensor control is necessary and desired [219]?
Alienation from nature	What are the consequences of losses of daylight/weather/seasonal effects due to urban densification, loss of daylight recreation areas, daylight restriction due to high buildings etc. on mood, health and quality of life [69,220,221,222,223]?
Role of view	Low daylight exposure and daylight deprivation usually also means deprivation from a view. This could have additional and far-reaching negative consequences, although research to date is scarce [223].

**Table 7:** Gaps in knowledge related to measurement tools and methods.

Automated integration of daylight and electric light in buildings	Lack of sophisticated and automated integration of daylight and electric light in buildings and algorithms to detect the two separately (this would also support reduction of lighting derived energy costs)
Monitoring spectral irradiance	Lack of validated, commercially available and affordable wearable devices for continuously monitoring spectral irradiance (at eye level). This could also be used as a “dosimeter” in research, therapies, living/working spaces and for lifestyle applications.
Tools for circadian phase assessments	Lack of practical means to make circadian phase assessments in daily life, the clinic, and elsewhere (comparing electric and daylight conditions).
Tools for mental health evaluations	Long-term daily mental health evaluations; approaches developed so far are wrist-worn diaries with visual analogue scales (also via mobile derived apps).
Standard operation procedures for the use of daylight treatment and daylight exposure (with regards to non-visual light responses)	Lack of standard operation procedures (SOPs) and definitions of daylight treatment responses for different individuals and patients. There is a need for large-scale field studies in schools, institutions (e.g., hospitals, prisons, care homes), shift- and non-shift workplaces, people working underground and people frequently traveling across times zones (with different overlay stays) using the same SOPs.
Norms and metrics	No (internationally accepted) consensus on the parameters to be measured and reported, and at what level of accuracy the monitoring tools can achieve this (see Section 2.3). A recent tutorial paper summarises the most important requirements [224]. One question that also arises: What is a suitable light metric to measure “naturalness” of light? How can we compare electric light sources to daylight? Some existing official metrics are summarised in the most recent international CIE standard, even though the D65/D55 parameters do not reflect the spectrum of daylight [225] and need to be revised to incorporate seasonal and geographical changes.
Large scale lighting digitalisation	Lack of large scale/practical biomedical digital techniques to design, monitor, predict and validate individually tailored daylight exposure/electric light regimens.

**Table 8:** Short-term objectives in daylight research.

Criteria	Define criteria for (day) light measures (see e.g., the new CIE standard S026). Universally agree to use this new standard, which is facilitated by the CIE-S-026-EDI-Toolbox-beta version E1.051.xls based on Ref. [228] and a more recent Tutorial [224].
Daytime biomarkers for physiology and behaviour	Replicate physiological, cognitive and emotional outcome (bio-) markers, and validate more than once in different laboratories, in order that they be implemented as reliable markers for describing the effect of daylight on physiology and behaviour.
Devices to monitor daylight	Develop robust, validated and commercially affordable devices to monitor spectral daylight exposure (representing light exposure at eye level in a vertical direction) along with temperature and humidity continuously indoors and outdoors. The need to calibrate and correct outputs from light recordings has been shown mostly for wrist worn devices [229,230].
Application in different populations	The questions discussed in Section 2 and Section 3 additionally need to be carried out in men and women, different ages (children, elderly) and ethnic groups, monitored across different environments (school/workplace/home); seasons; as well as in different patient groups (such as disorders of the nervous system, eyes).
Standard/exemplary data sets	Collect and evaluate different data sets to assess how much and which qualities of daylight different groups of individuals/patients receive at their work- and living places (24/7).
Status quo in real life	Define the status quo in daylight: irradiance measurements (including melanopic irradiance), interviews/focus groups with target populations (e.g., care home residents, prison inmates, dermatologists, ophthalmologists, shift- and night workers, tourist industry, people working underground (at e.g., train stations, miners), and related disciplines.

**Table 9:** Long-term objectives in daylight research.

Laboratory and field studies	Do prospective longitudinal and multicentre studies using the established SOPs under field and laboratory conditions in a sufficiently large sample, performed in both males and females.
Combined day- and electric light interventions	Compare light interventions with daylight exposure, and not only electric light OR daylight but also the (dynamic) mixture of both should be studied, since this is the norm. Additionally, a suitable scheduled length of daily light exposure (daily accumulation) requires monitoring over a long duration (chronic/seasonal light exposure).
Guidelines/recommendations for research and clinical studies	Propose guidelines/recommendations on how to set-up daylight research studies and clinical trials with daylight interventions.
Guidelines/recommendations for short- and long-term interventions	Propose guidelines/recommendations on how to measure/monitor daylight interventions and related physiological and behavioural outcomes over time.
Tools for inter-individual differences and circadian phase	Develop reliable tools to assess inter-individual differences and internal circadian phase for users and practitioners.
Instruments for light monitoring in the field	Further validate instruments which monitor individual light and colour perception/preferences under daylight conditions in the field.
Daylight recommendations for task and biological requirements	Develop daylight recommendations, which relate to both task requirements and physiological/psychological aspects.
Education	Educate professionals, government policy makers, and the public in ‘light hygiene’ e.g., sufficient vs. inadequate vs. too much daylight exposure.
Architecture/building science	Early stage planning of building/window positioning optimised for better daylight exposure and spatial-temporal modulation. Develop real mixed electric light/daylight simulation tools. Multimodal approach for optimised light exposure, heat emission, noise, air quality.
Database	Create an open access daylight database (wiki/online depository).
Modelling	Develop mathematical models that help predict biologically appropriate daylight exposure (e.g., characteristics such as timing, dose, spectral composition and light exposure pattern) for promoting circadian entrainment, sleep, performance and health in addition to the required standards for visual function at the level of the individual as well as in different populations.
